# The Role of Fucoidans Isolated from the Sporophylls of *Undaria pinnatifida* against Particulate-Matter-Induced Allergic Airway Inflammation: Evidence of the Attenuation of Oxidative Stress and Inflammatory Responses

**DOI:** 10.3390/molecules25122869

**Published:** 2020-06-22

**Authors:** Kalahe Hewage Iresha Nadeeka Madushani Herath, Hyo Jin Kim, Areum Kim, Chung Eui Sook, Boo-Yong Lee, Youngheun Jee

**Affiliations:** 1 Department of Veterinary Medicine and Veterinary Medical Research Institute, Jeju National University, Jeju 63243, Korea; madushaniherath001@gmail.com (K.H.I.N.M.H.); orange5687@naver.com (A.K.); 2 Department of Food Bioengineering, Jeju National University, 102 JeJudaehakno, Jeju 63243, Korea; jyouding@gmail.com; 3 Haewon Biotech, Inc., Seoul 110020, Korea; biosook@hanmmail.net; 4 Department of Biomedical Science, CHA University, Seongnam 463-836, Korea; bylee@cha.ac.kr; 5 Interdisciplinary Graduate Program in Advanced Convergence Technology & Science, Jeju National University, Jeju 63243, Korea

**Keywords:** particulate matter, fucoidan, asthma, airway, inflammatory response, mucus hypersecretion

## Abstract

Ambient particulate matter (PM) is a critical environment pollutant that promotes the onset and aggravation of respiratory diseases such as asthma through airway inflammation and hypersecretion of mucus. In this study, we aimed to identify the effects of fucoidans isolated from sporophylls of *Undaria pinnatifida* on asthma symptoms such as the inflammatory response and mucus secretion using a mouse model. Balb/c mice, intraperitoneally sensitized with ovalbumin (OVA, 10 μg) dissolved in 200 µL saline and 2 mg Al(OH)_3_, were exposed to PM (5 mg/m^3^) for 7 consecutive days. In parallel, along with PM exposure, we orally administrated fucoidans (100, 400 mg/Kg) or prednisone (5 mg/Kg), an anti-inflammatory drug. We found that oral administration of fucoidans significantly attenuated PM-induced lipid peroxidation and infiltration of inflammatory cells like F4/80^+^ macrophages, Gr-1^+^ granulocytes, and CD4^+^ T lymphocytes. Fucoidans also attenuated the level of PM-exacerbated IL-4, a primitive cytokine released in Th2 mediated eosinophilic asthma. This further suppressed mast cell activation, degranulation and IgE synthesis of PM exposed mice. Interestingly, fucoidans attenuated PM-exacerbated mucus hypersecretion and goblet cell hyperplasia. Therefore, our results suggest that fucoidans are effective at alleviating PM-exacerbated allergic asthma symptoms by attenuating the airway inflammatory response and mucus hypersecretion.

## 1. Introduction

Particulate matter (PM) is a major air pollutant that has gained considerable attention over the last few years due to rapid urbanization and industrialization. The pathogenicity of PM depends on its composition and size [1]. The toxic components of PM, such as heavy metals, polycyclic aromatic hydrocarbons, and organic carbon and biological materials, vary according to the emission source and season [2]. Also, PM with a diameter of <2.5 μm (PM_2.5_) can easily remain airborne and penetrate into the deep airways when inhaled. Polycyclic aromatic hydrocarbons and heavy metals in PM can induce reactive oxygen species (ROS) production and inflammation in the airway, further exacerbating pre-existing asthma symptoms or triggering the onset of asthma [3].

Asthma is a heterogeneous inflammatory disease of the airways of the lungs that is characterized by an influx of inflammatory cells followed by airway obstruction, airway hyperresponsiveness, and hypersecretion of mucus [4]. The classical form of asthma is considered to be mediated by T helper cell type 2 (Th2) cells and is characterized by eosinophilic inflammation, immunoglobulin (Ig) E reactivity, and the predominance of Th2 cytokines such as interleukin (IL)-4, IL-5, and IL-13 [5]. Moreover, PM has been identified to activate Th17-mediated immune responses along with the overproduction of cytokine IL-17a, which further promotes Th2-mediated eosinophilic inflammation as well as the influx of neutrophils to airways, further worsening asthma symptoms [5]. Current treatments for asthma focus mainly on suppressing the inflammatory response. Corticosteroids are known as effective controllers of asthma as they suppress inflammation, edema, and mucus hypersecretion [6]. However, some patients do not benefit from anti-inflammatory drugs, and, moreover, anti-inflammatory drugs like corticosteroids can cause irreversible changes in the lungs, which may lead to adverse effects with long-term use [7]. Therefore, there is an unmet need for alternative therapies based on natural products for treating asthma.

Fucoidans are sulphated polysaccharides isolated from many species of brown algae. They have been identified to possess a wide range of biological activities such as anti-inflammation, anti-allergic, anti-cancer, and anti-thrombotic effects [8,9,10]. In this study, we evaluated fucoidans isolated from the sporophylls of *Undaria pinnatifida* (*U. pinnatifida*), an edible brown algae that is native to the cold temperate seas of Korea, Japan, and China [11]. It is an invasive species with high tolerance for light, temperature, and salinity, and it has a high reproductive rate, producing spores all around the year [12]. Sporophylls located at the root part of this alga have been identified as a plentiful source of fucoidans [13]. Moreover, fucoidans isolated from sporophylls of *Undaria pinnatifida* have garnered much attention due to their richness in sulfate and L-fucose contents compared with other fucoidans [14]. According to Kim et al. (2010), the monosaccharide composition of fucoidans from *U. pinnatifida* sporophylls was as follows: 64.4 ± 6.0% fucose, 31.9 ± 4.7% galactose, and 3.6 ± 1.3% mannose [14]. The proportion of sulfates was 31.7 ± 2.2% [14]. Further studies discovered that fucoidans from *U. pinnatifida* sporophylls attenuated ROS formation and the secretion of the inflammatory cytokines TNF-α, MCP-1, and IL-6 in 3T3-L1 adipocytes [15]. Moreover, fucoidans from *U. pinnatifida* suppressed the secretion of inflammatory cytokines IL-1β and tumor necrosis factor (TNF)-α via attenuating mitogen-activated protein kinases and oxidative stress in lipopolysaccharide (LPS) induced RAW264.7 cells [16]. Maruyama et al. [17], previously discovered that fucoidans isolated from *U. pinnatifida* attenuated the Th2 immune response by attenuating the levels of Th2 IL-4, IL-5, and IL-13 in broncho alveolar lavage (BAL) fluid as well as the immunoglobulin E (IgE) level in the serum of ovalbumin-induced mice [17]. However, the study by Maruyama et al. [17] was limited to the Th2 inflammatory process of *U. pinnatifida* isolated fucoidans by targeting Th2 cytokines. Thus, their role on cellular participants in the initial recognition and maintenance phase of this immune response is still elusive. The findings of Mayurama et al. [17], suggest that fucoidans from *U. pinnatifida* sporophylls might also be therapeutically effective against PM-exacerbated allergic inflammatory responses. Based on this hypothesis, we examined the therapeutic effect of fucoidans from *U. pinnatifida* sporophylls on a mouse model of allergic airway inflammation. In this study, we investigated the therapeutic potential of fucoidans isolated from the *U. pinnatifida* sporophylls regarding the attenuation of PM-exacerbated allergic inflammatory responses. We focused on PM-induced initial oxidative stress, the activation of inflammatory cells, and, thereby, immunoglobulin production, histopathological changes and mucus secretion.

In our animal model, we exposed ovalbumin (OVA)-sensitized mice to nebulized PM to mimic human PM exposure in the atmosphere. This setup strengthened the similarity between our mouse model and actual human pathophysiology of PM-exacerbated allergic airway inflammation and hence increased the relevance of our model. Supporting these data, fucoidans decreased OVA-induced eosinophil influx into allergic sites via blocking L-selectin, which is crucial for γδ T lymphocyte activation and its migration to inflammatory sites [18].

## 2. Results

### 2.1. The Effect of Fucoidan on the Levels of 8-OHdG and Malondialdehyde (MDA) in the Serum and Lungs

**Figure 1:**
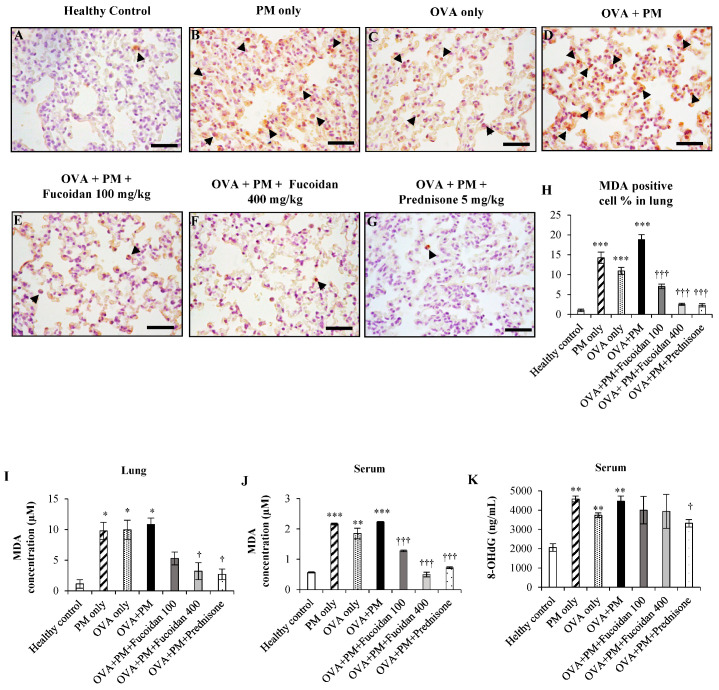
The effect of fucoidans on the levels of MDA and 8-OHdG in the lungs and serum. The lung (**A**–**G**) MDA positive cells, (**H**) percentages of MDA positive cells and (**I**) the MDA level are presented. Serum (**J**) MDA and (**K**) 8-OHdG levels are presented. Values are expressed as means ± SEM (*n* = 3). * (*p* < 0.05), ** (*p* < 0.05), *** (*p* < 0.05) represent significant increases compared with the healthy control, and † (*p* < 0.05) ††† (*p* < 0.0005) represent significant decreases compared with the OVA + PM group.

Exposure to PM initially triggers oxidative stress, and the oxidative stress further leads to lipid peroxidation. We analyzed the level of MDA, a secondary compound formed by the peroxidation of unsaturated fatty acids. We evaluated the MDA positive cell percentage in the lungs (Figure 1A–H). PM exposure as well as OVA significantly (*p* < 0.0005) enhanced the MDA positive cell percentage in PM only, OVA only, and OVA + PM compared to a healthy control. Fucoidans dose-dependently attenuated the MDA positive cell percentage in the lungs compared with OVA + PM.

In parallel, we analyzed the level of MDA in the lung homogenate (Figure 1I). PM exposure, as well as OVA, significantly (*p* < 0.05) enhanced the level of MDA (increased by 88.4% in PM only, by 88.6% in OVA, by 89.5% in OVA + PM) compared with a healthy control. However, fucoidan attenuated the PM-exacerbated level of MDA dose-dependently in the lungs. We observed a 51.2% reduction with low dose (100 mg/kg) fucoidan treatment and a 70.4% reduction (*p* < 0.05) with high dose (400 mg/kg) fucoidan treatment compared with OVA + PM. A high dose of fucoidan was more or similarly effective to prednisone, the positive control, at attenuating the PM-exacerbated MDA level in this study.

We further analyzed the MDA level in serum. We noticed that PM exposure significantly enhanced the MDA level in serum (increased by 74.6% in both PM only and OVA + PM, both *p* < 0.005) compared with the healthy control (Figure 1J). Interestingly, fucoidan dose-dependently attenuated the PM-exacerbated level of MDA in serum. We observed a 44.8% reduction (*p* < 0.0005) with low-dose (100 mg/kg) fucoidan treatment and a 78.0% reduction (*p* < 0.0005) with high-dose (400 mg/kg) fucoidan treatment compared with OVA + PM.

We also examined the level of 8-OHdG in murine serum (Figure 1K). As in the case of MDA analysis, we observed a significant increase in the serum 8-OHdG level in OVA only (by 81.4%, *p* < 0.005), PM only (by 122.2%, *p* < 0.005), and OVA + PM groups (by 116.8%, *p* < 0.005) compared with the healthy control. Although not significant, fucoidans attenuated the 8-OHdG level in serum compared with the OVA + PM group (reduced by 11.8% with both fucoidan doses). In the meantime, prednisone attenuated the 8-OHdG level in serum by 25.7% (*p* < 0.05) compared with OVA + PM. These results suggest that fucoidans and prednisone attenuate oxidative stress induced by PM through different signaling pathways and hence show different efficacies with different measurement methods.

### 2.2. Fucoidans Attenuated PM-Exacerbated Airway Hyper Responsiveness and Inflammatory Cell Infiltration in Broncho Alveolar Lavage Fluid (BALF)

**Figure 2:**
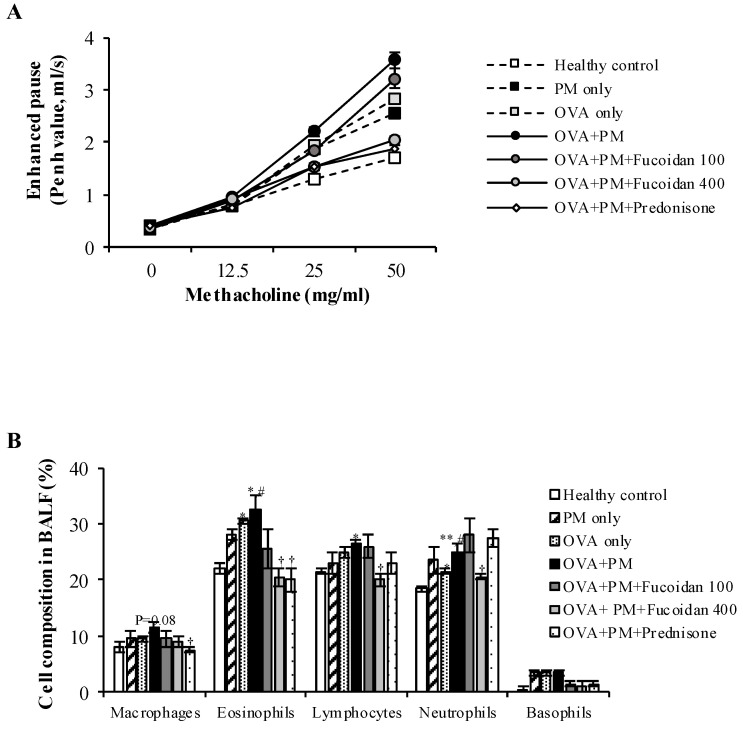
The effect of fucoidans on PM-exacerbated airway hyper-responsiveness and differential cells in the broncho alveolar lavage fluid (BALF). (**A**) Airway hyper-responsiveness is shown as Penh values in response to increasing concentrations of methacholine (0–50 mg/mL). (**B**) The number of inflammatory cells in the BALF. Values are expressed as means ± SEM of at least two individual experiments (*n* = 6). * (*p* < 0.05), ** (*p* < 0.005), represent significant increases compared with the healthy control, and † (*p* < 0.05), †† (*p* < 0.005), represent significant decreases compared with the OVA + PM group. # represents a significant increase compared with OVA only.

We evaluated the effect of fucoidans on airway resistance (Penh value) in response to methacholine inhalation in OVA-sensitized, PM-exposed mice (Figure 2A). The PM only, OVA only, and OVA + PM groups showed significant (*p* < 0.0005) increases in airway hyper-responsiveness compared with the healthy control when exposed to a high concentration of methacholine (50 mg/mL). Moreover, PM exacerbated OVA-induced airway hyper-responsiveness (by 20.7%, *p* < 0.05). More importantly, the group that received a high dose of fucoidans had markedly attenuated (by 43.0%, *p* < 0.0005) PM-exacerbated airway hyper-responsiveness compared with OVA + PM (with 50 mg/mL methacholine). A high dose of fucoidans was similarly effective to prednisone at attenuating PM-exacerbated airway hyper-responsiveness.

To determine whether fucoidans attenuate the PM-exacerbated airway inflammatory response, we assessed the number of various inflammatory cells in the BALF (Figure 2B). Groups that received a high dose of fucoidans had significantly (*p* < 0.05) attenuated PM-exacerbated infiltration of inflammatory cells; eosinophils, neutrophils, and lymphocytes in BALF. Interestingly, a high dose of fucoidans effectively attenuated neutrophil infiltration more than prednisone did. Although not significant, the fucoidans dose-dependently attenuated the PM-exacerbated macrophage and basophil counts in BALF.

### 2.3. Pathological Observations in Mouse Trachea and Lungs

**Figure 3:**
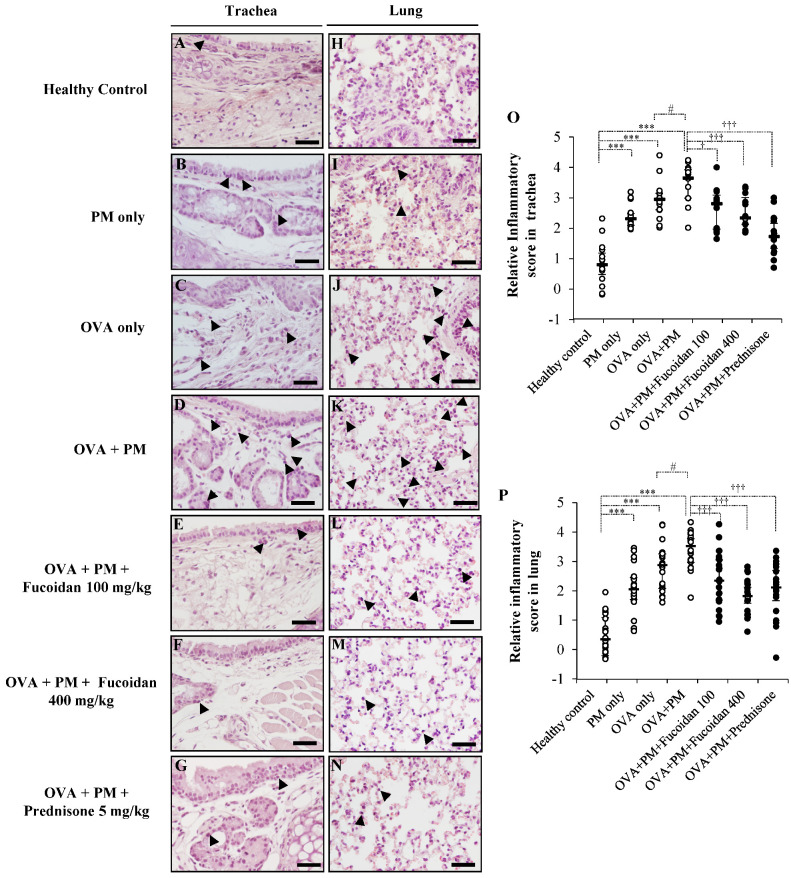
PM-aggravated histological changes in vivo and the effect of fucoidans on them. Representative images of H&E stained (**A**–**G**) trachea and (**H**–**N**) lung sections are shown. (**A**,**H**) Healthy control, (**B**,**I**) PM only, (**C**,**J**) OVA only, (**D**,**K**) OVA + PM, (**E**,**L**) OVA + PM + Fucoidans (100 mg/Kg), (**F**,**M**) OVA + PM+ Fucoidans (400 mg/Kg), (**G**,**N**) OVA + PM + Prednisone (5 mg/Kg). The infiltration of inflammatory cells is indicated with arrowheads. Scatter plots showing the median and interquartile range of the inflammatory scores for tracheal sections (**A**–**G**) are shown in (**O**) and those for lung sections (**H**–**N**) are shown in (**P**). The degree of peri-bronchial and perivascular inflammation of lung and tracheal sections (at least 6 sections/mice, *n* = 4) from each group were separately evaluated on a subjective scale as follows: 0, normal; 1, few inflammatory cells infiltrated; 2, infiltration of inflammatory cells one cell layer deep; 3, infiltration of inflammatory cells 2–4 cell layers deep; and 4, a ring of inflammatory cells more than four cell layers deep. The scale bar of (**A**–**N**) is 25 µm). We added random jitter in the vertical direction to avoid overlapping data points. The error bars represent the interquartile range and median. *** (*p* < 0.0005) represents a significant increase compared with the healthy control, and † (*p* < 0.05), ††† (*p* < 0.0005) represents a significant decrease compared with the OVA + PM group. # represents a significant increase compared to OVA only.

To further verify the potential of fucoidans to attenuate PM-exacerbated inflammatory responses, we stained mouse trachea and lung sections with hematoxylin and eosin (H&E) and observed the histopathological changes (Figure 3) along with the grading of inflammatory activity (Figure 3O (trachea), Figure 3P (lungs)). The infiltration of inflammatory cells in the OVA + PM group was higher than that in the healthy control, PM only, and OVA only groups in the trachea as well as in the lungs. The groups that received a high dose of fucoidans attenuated PM-exacerbated inflammatory cell infiltration in the trachea (Figure 3F,O) and lungs (Figure 3M,P). Interestingly, fucoidans were as effective as prednisone at attenuating PM-exacerbated inflammatory cell infiltration.

### 2.4. Fucoidans Attenuated the Infiltration of Eosinophils in the Trachea and Lungs

**Figure 4:**
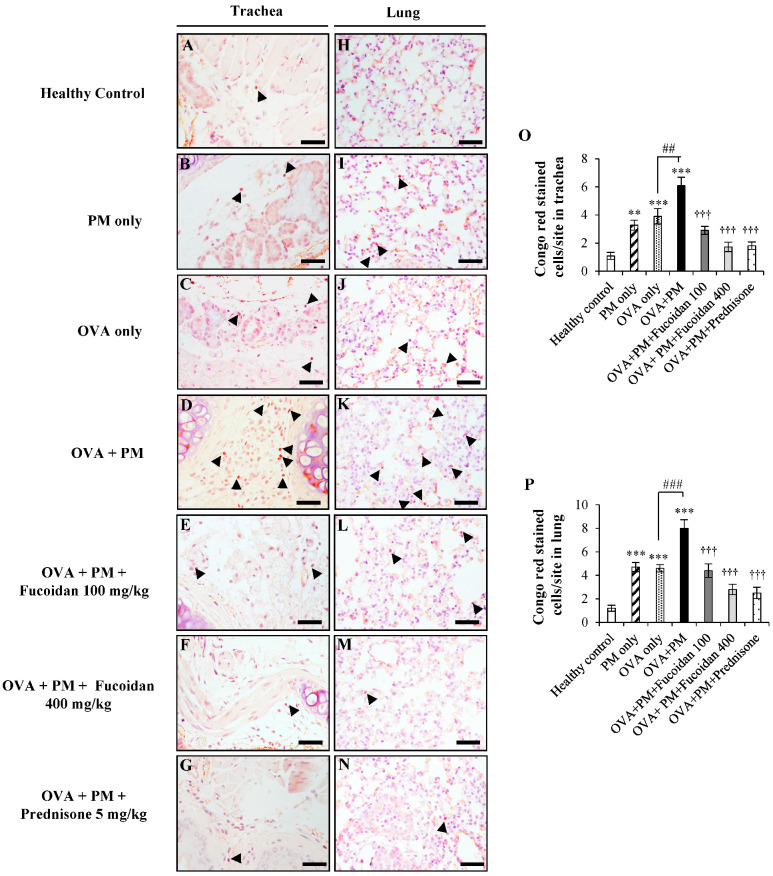
Congo red staining of the trachea (**A**–**G**) and lungs (**H**–**N**). (**A**,**H**) Healthy control, (**B**,**I**) PM only, (**C**,**J**) OVA only, (**D**,**K**) OVA + PM, (**E**,**L**) OVA + PM + Fucoidans (100 mg/Kg), (**F**,**M**) OVA + PM + Fucoidans (400 mg/Kg), (**G**,**N**) OVA + PM + Prednisone (5 mg/Kg). (**O**) Number of Congo red stained cells per site in the trachea, (**P**) Number of Congo red stained cells per site in the lungs. Values are expressed as means ±SEM (*n* = 3). ** (*p* < 0.005), *** (*p* < 0.0005) represent significant increases compared with the healthy control, and ††† (*p* < 0.0005) represents a significant decrease compared with the OVA + PM group. ## (*p* < 0.005), ### (*p* < 0.0005) represents a significant increase compared with the OVA only group. The scale bar of (**A**–**N**) is 25 µm.

Among the infiltrated inflammatory cells, an influx of eosinophils was particularly observed in asthma conditions, establishing pulmonary inflammation and airway hyper-responsiveness. To evaluate the effect of fucoidans on eosinophil infiltration, we stained the trachea and lung sections using Congo red. Representative images of lung and trachea lesions are shown in Figure 4. PM increased eosinophil infiltration in the trachea of OVA-sensitized mice by 500.0% compared with the healthy control (Figure 4A,O; *p* < 0.0005).

PM and OVA alone also enhanced eosinophil infiltration in the trachea, though to lesser levels than in OVA + PM, and OVA + PM exacerbated the eosinophil infiltration more (by 56.4%, *p* < 0.005) compared with the OVA only group (Figure 4C,O). However, fucoidan treatments clearly attenuated PM-exacerbated eosinophil infiltration in the trachea in a dose response manner compared with the OVA + PM group (suppression by 52.0% and 72.0% for low- and high-dose fucoidan treatments, respectively; Figure 4D,O). Prednisone (Figure 4G,O) attenuated eosinophil infiltration in the trachea, as expected.

Similarly, the highest infiltration of eosinophils was observed in the OVA + PM group (Figure 4K,P) in the lungs compared with the healthy control (by 566.6%, *p* < 0.0005) and with OVA only (74.0%, *p* < 0.005). However, fucoidan dose-dependently attenuated eosinophil infiltration in the lungs (suppression by 45.0% and 65.0% for low- and high-dose fucoidan treatments, respectively; Figure 4M,P). Furthermore, high-dose fucoidan treatment was as effective as prednisone treatment (Figure 4N,P) for attenuating the PM-exacerbated eosinophil count in mouse lungs.

### 2.5. Fucoidans Attenuated the Infiltration of Gr-1^+^ Cells in the Trachea and Lungs

**Figure 5:**
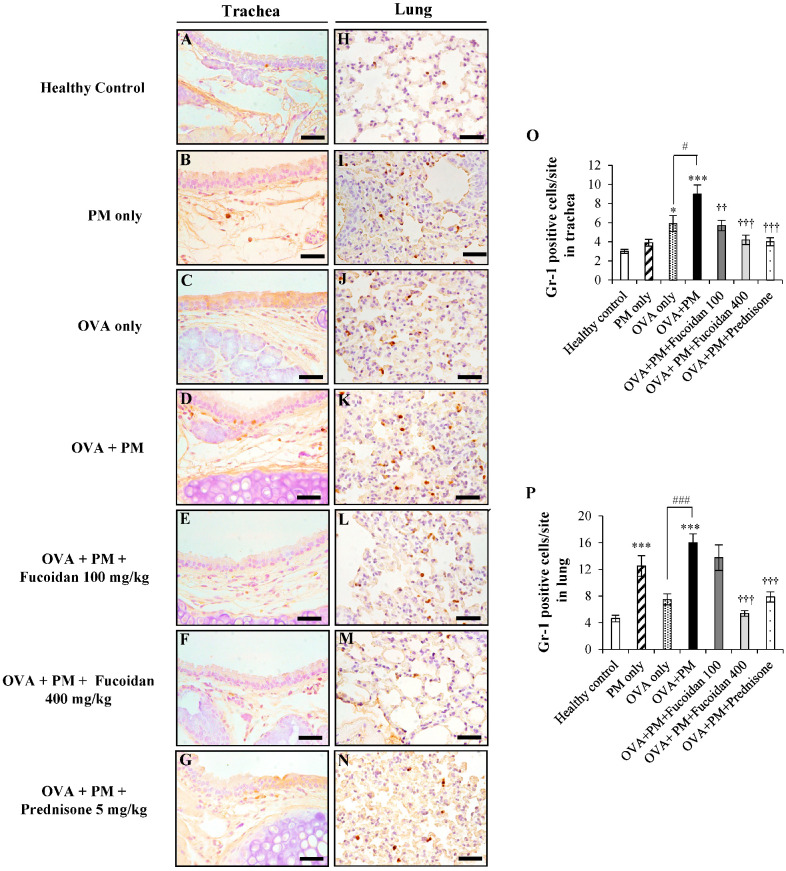
Immunohistochemical analysis of Gr-1^+^ cells in the trachea (**A**–**G**) and lungs (**H**–**N**). (**A**,**H**) Healthy control, (**B**,**I**) PM only, (**C**,**J**) OVA only, (**D**,**K**) OVA + PM, (**E**,**L**) OVA + PM + Fucoidan (100 mg/Kg), (**F**,**M**) OVA + PM + Fucoidan (400 mg/Kg), (**G**,**N**) OVA + PM + Prednisone (5 mg/Kg). (**O**) Gr-1^+^ cells per site in the trachea, (**P**) Gr-1^+^ cells per site in the lungs. Values are expressed as means ± SEM (*n* = 3). * (*p* < 0.05), *** (*p* < 0.0005) represent significant increases compared with the healthy control, and †† (*p* < 0.005), ††† (*p* < 0.0005) denote significant decreases compared with the OVA + PM group. # (*p* < 0.05), ### (*p* < 0.0005) denote significant increases (*p* < 0.05) compared with the OVA only group. The scale bar of (**A**–**N**) is 25 µm.

We further confirmed the infiltration of granulocytes such as eosinophils and neutrophils, predominant players in airway inflammation in PM-exacerbated allergic airway inflammation by performing immunohistochemical staining. Representative images of the immunohistochemical analysis of Gr-1^+^ cells in the trachea and lungs of the PM-exacerbated murine model of allergic airway inflammation are shown in Figure 5.

The OVA + PM group (Figure 5D,O) enhanced the influx of granulocytes in the trachea significantly (by 200.0%, *p* < 0.0005) compared with the healthy control. PM exacerbated Gr-1^+^ cell infiltration by 52.5% in the trachea of OVA-sensitized mice compared with treatment with OVA alone (Figure 5C,O). However, fucoidan treatments attenuated the PM-exacerbated influx of Gr-1^+^ cells in the trachea. We observed a 36.6% reduction with low-dose fucoidan treatment (Figure 5E,O) and a 53.3% reduction with high-dose fucoidan treatment (Figure 5F,O) compared with OVA + PM. Prednisone also attenuated the granulocyte influx by 55.5% compared with OVA + PM (Figure 5G,O).

Then, we evaluated the effect of fucoidans on the infiltration of granulocytes in the lungs of asthmatic mice. We observed a 247.8% increase in granulocyte infiltration in OVA + PM (Figure 5K,P) compared with the healthy control (Figure 5H,P). In the meantime, PM exacerbated OVA-induced granulocyte infiltration compared with OVA only (by 113.3%, *p* < 0.0005). However, when treated with a high dose of fucoidans (Figure 5M,P), there was a significant reduction of granulocytes infiltration in lung compared to OVA + PM (by 66.4%, *p* < 0.0005). More importantly, fucoidans was more effective than prednisone at attenuating PM-exacerbated granulocyte infiltration in the lungs in OVA-sensitized mice.

### 2.6. Fucoidans Attenuated F4/80^+^ Macrophage Infiltration in the Trachea and Lungs

**Figure 6:**
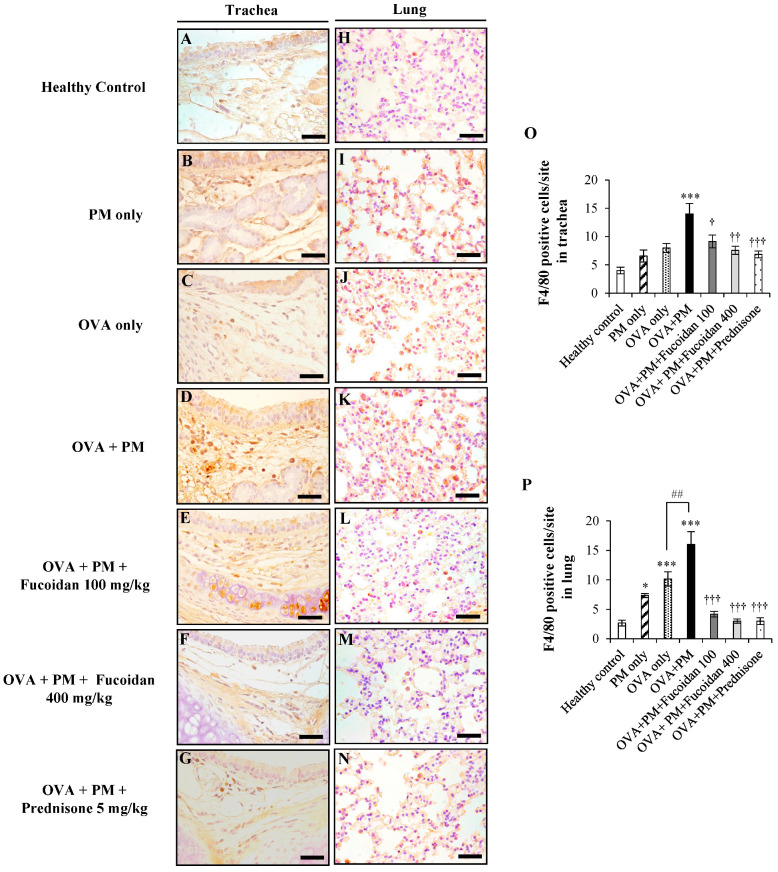
Immunohistochemical analysis of F4/80^+^ macrophage cells in the trachea (**A**–**G**) and lungs (**H**–**N**). (**A**,**H**) Healthy control, (**B**,**I**) PM only, (**C**,**J**) OVA only, (**D**,**K**) OVA + PM, (**E**,**L**) OVA + PM + Fucoidan (100 mg/Kg), (**F**,**M**) OVA + PM + Fucoidan (400 mg/Kg), (**G**,**N**) OVA + PM + Prednisone (5 mg/Kg). (**O**) Number of F4/80^+^ cells per site in the trachea, (**P**) Number of F4/80^+^ cells per site in the lungs. Values are expressed as means ± SEM (*n* = 3). * (*p* < 0.05), *** (*p* < 0.0005) represent significant increases compared with the healthy control, and † (*p* < 0.05), †† (*p* < 0.005), ††† (*p* < 0.0005) denote significant decreases compared with the OVA + PM group. ## denotes a significant increase (*p* < 0.005) compared with the OVA only group. The scale bar of (**A**–**N**) is 25 µm.

Macrophages are abundant immunocytes in the lungs that are involved in the progression of the inflammatory response during the pathogenesis of allergic airway inflammation. Hence, the dysfunction of macrophages is of great interest in asthma therapies. To study the effect of fucoidans on macrophages, we performed an immunohistochemical analysis of F4/80^+^ cells in the trachea and lung of the PM-exacerbated murine model of allergic airway inflammation (Figure 6). The OVA + PM group showed clearly enhanced macrophage infiltration in the trachea (+250.0%, *p* < 0.0005) compared with the healthy control. However, fucoidans significantly reduced macrophage infiltration in the trachea compared with the OVA + PM group (by 34.7%, *p* < 0.05 at a low dose; by 45.7%, *p* < 0.0005 at a high dose).

The immunohistochemical analysis also revealed an increase of 515.3% (*p* < 0.0005) in F4/80^+^ macrophage infiltration in the lungs of PM + OVA mice compared with the healthy control. Moreover, PM exacerbated macrophage infiltration in the lungs of OVA-sensitized mice (OVA + PM, Figure 6K,P) compared with treatment with OVA only (Figure 6J,P) (by 58.4%, <0.005). However, mice treated with a low dose (Figure 6L,P) and a high dose of fucoidans (Figure 6M,P) significantly attenuated PM-exacerbated macrophage infiltration in the lungs (by 74.3% and 81.2% respectively, both *p* < 0.0005) compared with OVA + PM. Furthermore, high-dose fucoidan treatment was more or similarly effective to prednisone for attenuating macrophage infiltration.

### 2.7. Fucoidans Attenuated CD4^+^ T Cell Infiltration in the Trachea and Lungs

**Figure 7:**
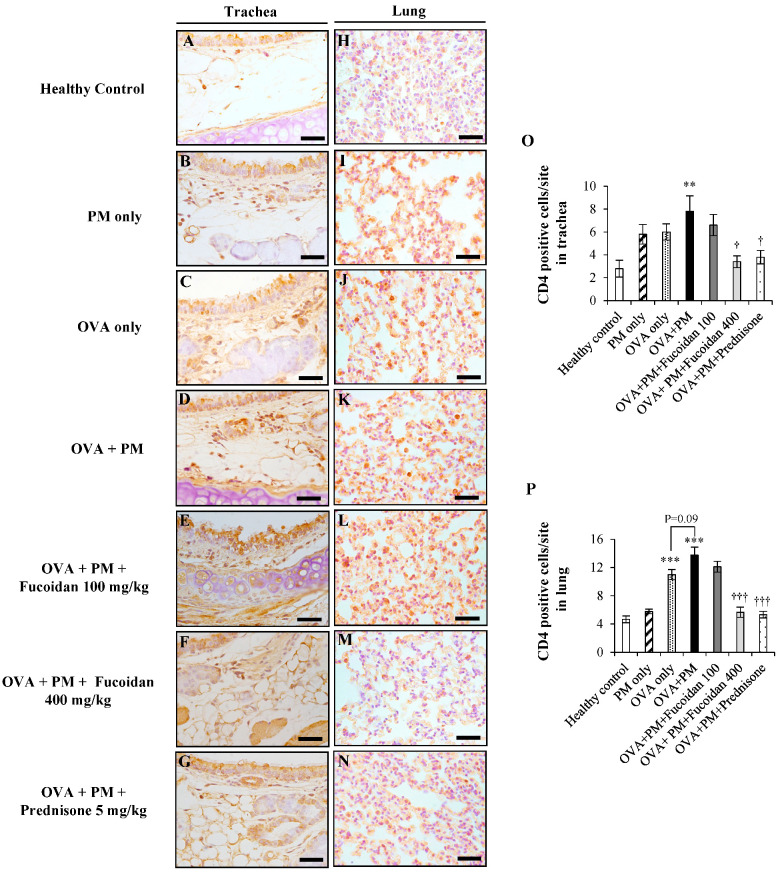
Immunohistochemical analysis of CD4^+^ T cells in the trachea (**A**–**G**) and lungs (**H**–**N**). (**A**,**H**) Healthy control, (**B**,**I**) PM only, (**C**,**J**) OVA only, (**D**,**K**) OVA + PM, (**E**,**L**) OVA + PM + Fucoidans (100 mg/Kg), (**F**,**M**) OVA + PM + Fucoidans (400 mg/Kg), (**G**,**N**) OVA + PM + Prednisone (5 mg/Kg). (**O**) Number of CD4^+^ T cells per site in the trachea, (**P**) number of CD4^+^ T cells per site in the lungs. Values are expressed as means ±SEM (*n* = 3). ** (*p* < 0.005), *** (*p* < 0.0005) represent significant increases compared with the healthy control, and † (*p* < 0.05), ††† (*p* < 0.0005) denote significant decreases compared with the OVA + PM group. The scale bar of (**A**–**N**) is 25 µm.

CD4^+^ T cells are at the forefront in the pathogenesis of allergic airway inflammation as they specifically identifying allergens and initiate an inflammatory response in the respiratory system. We observed a marked increase (by 64.1%, *p* < 0.005) in CD4^+^ T cells in OVA + PM (Figure 7D,O) compared with the healthy control (Figure 7A,O). In contrast, CD4^+^ T cell infiltration was reduced substantially in a dose-dependent response manner with fucoidan treatments compared with OVA + PM: by 15.4% at a low dose (Figure 7E,O) and by 56.4% (*p* < 0.05) at a high dose (Figure 7F,O).

We also evaluated the effect of fucoidans on the infiltration of CD4^+^ T cells in the lungs of OVA + PM mice. We observed a 197.8% increase in CD4^+^ T cell infiltration in OVA + PM (Figure 7K,P) compared with the healthy control (Figure 7H,K). In the meantime, PM exacerbated OVA-induced CD4^+^ T cell infiltration compared to treatment with OVA only (by 24.5%, *p* < 0.09). However, when treated with a high dose of fucoidan (Figure 7M,P), there was a significant reduction in CD4^+^ T cell infiltration in the lungs compared with OVA + PM (by 59.1%, *p* < 0.0005). More importantly, prednisone had a similar effect to a high dose of fucoidans at attenuating PM-exacerbated CD4^+^ T cell infiltration in the lungs in OVA-sensitized mice.

### 2.8. Fucoidans Attenuated the Serum Level of Total IgE, an Antigen-Specific Antibody, in PM-Exposed and OVA-Sensitized Mice

**Figure 8:**
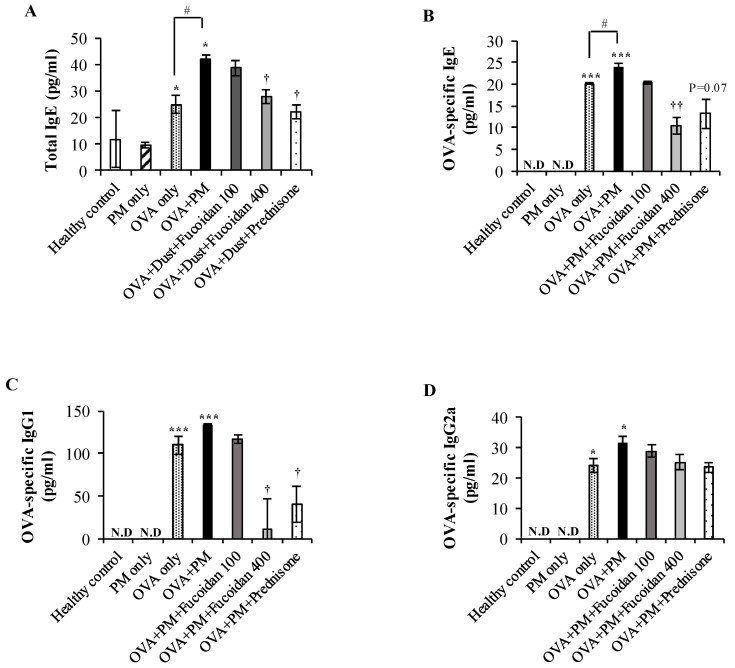
Effect of fucoidans on immunoglobulin levels in the serum. Levels of (**A**) total IgE, (**B**) OVA-specific IgE, (**C**) OVA-specific IgG1, and (**D**) OVA-specific IgG2a in serum. Values are expressed as means ± SEM (*n* = 3). * (*p* < 0.05), *** (*p* < 0.0005) represent significant increases compared with the healthy control, and † (*p* < 0.05), †† (*p* < 0.005) represent significant decreases compared with the OVA + PM group. # (*p* < 0.05) represents a significant increase compared with the OVA only group. N.D; not detected.

We evaluated the level of serum IgE, a key mediator of mast cell activation in allergic airway inflammation, since mast cells are activated when allergens bind to IgE (Amin, 2012). As shown in Figure 8A, the total IgE level was markedly increased (by 280.1%, *p* < 0.05) in OVA + PM compared with the healthy control. Also, PM augmented the serum IgE level in OVA-sensitized mice (by 68.3%, *p* < 0.05) compared with treatment with OVA only. Although fucoidans were not as effective as prednisone, high-dose fucoidan treatment also attenuated the PM-exacerbated serum IgE level considerably (by 33.2%, *p* < 0.005) compared with OVA + PM. We further evaluated the OVA-specific immunoglobulin level in serum. As shown in Figure 8B, the OVA-specific IgE level was markedly increased in OVA + PM compared with the healthy control (*p* < 0.0005) as well as in the OVA only group (by 18.90%, *p* < 0.05). A high dose of fucoidans significantly attenuated the OVA-specific IgE level (by 56.3%, *p* < 0.005) compared with OVA + PM and was more effective than prednisone. Moreover, OVA + PM moderately increased the OVA-specific IgG1 level (Figure 8C) compared with OVA only, and similarly to prednisone treatment, high-dose fucoidan treatment attenuated the OVA-specific IgG1 level significantly (by 90.0%, *p* < 0.05) compared with OVA + PM. However, fucoidans did not affect the OVA-specific IgG2a level (Figure 8D) in serum. These results suggest that PM exacerbated OVA sensitization.

### 2.9. Fucoidans Attenuated the Activation and Degranulation of Mast Cells in PM-Exposed and OVA-Sensitized Mice

**Figure 9:**
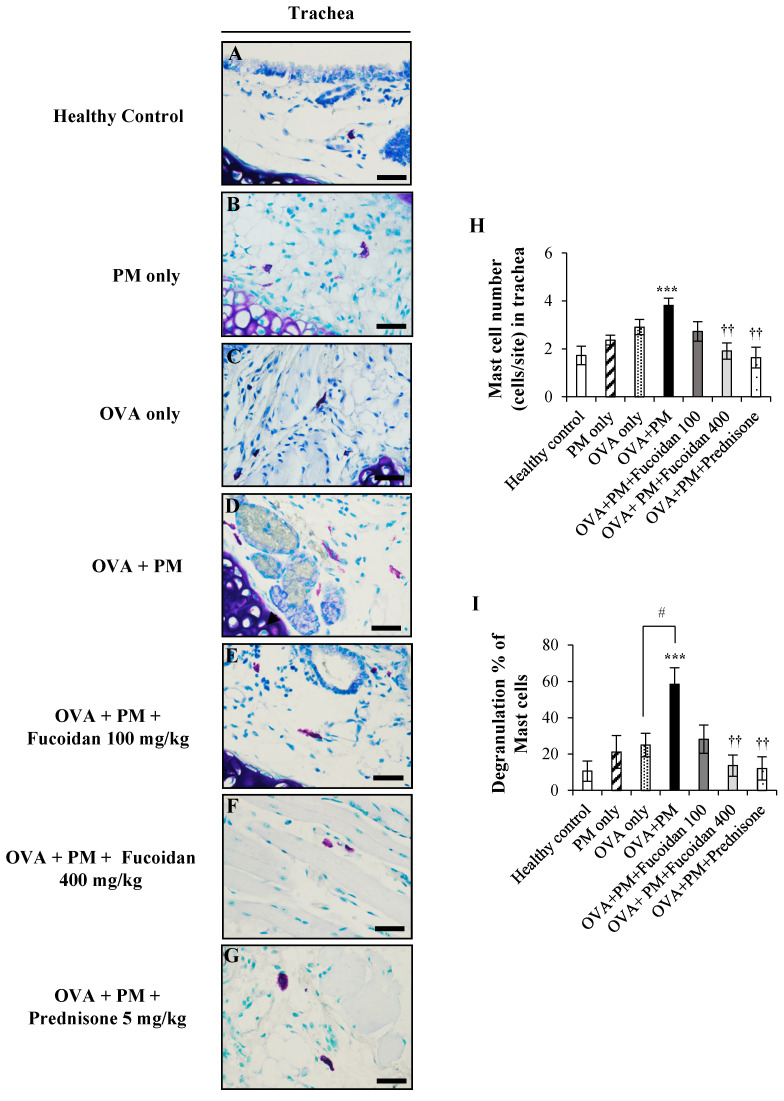
Toluidine blue staining of the trachea. (**A**) Healthy control, (**B**) PM only, (**C**) OVA only, (**D**) OVA + PM, (**E**) OVA + PM + Fucoidans (100 mg/Kg), (**F**) OVA + PM + Fucoidans (400 mg/Kg), (**G**) OVA + PM + Prednisone (5 mg/Kg). (**A**–**G**) Mast cell migration, (**H**) mast cell number per site, (**I**) mast cell degranulation percentage. Values are expressed as means ± SEM (*n* = 3). *** (*p* < 0.0005) represents a significant increase compared with the healthy control, and †† (*p* < 0.005) represents a significant decrease compared with the OVA + PM group. # (*p* < 0.05) represents a significant increase compared with the OVA only group. The scale bar of (**A**–**G**) is 25 µm.

We also examined the activation of mast cells that initiate and boost the airway inflammation in the pathogenesis of allergic asthma by toluidine blue staining. We observed a marked increase (by 123.5%, *p* < 0.0005) of activated mast cells in OVA + PM (Figure 9D,H) compared with the healthy control (Figure 9A,H). In contrast, mast cell activation was reduced substantially in a dose-dependent response manner with fucoidan treatments compared with OVA + PM: by 28.9% at a low dose (Figure 9E,H) and by 50.0% (*p* < 0.005)400 mg/kg at a high dose (Figure 9F,H).

Activated mast cells are known to undergo degranulation and release histamine and other vasoactive mediators. As shown in Figure 9I, OVA + PM showed the highest degranulation of mast cells compared with the PM and OVA only groups. PM exacerbated OVA-induced mast cell degranulation by 133.6% compared with treatment with OVA only. However, fucoidan treatments upturned the PM-induced degranulation of mast cells in the trachea: by 51.9% at a low dose and by 76.7% (*p* < 0.005) at a high dose, respectively. Interestingly, high dose fucoidan treatment was as effective as prednisone for attenuating PM-exacerbated degranulation of mast cells in OVA-sensitized mice.

### 2.10. Fucoidans Attenuated Goblet Cell Hyperplasia and Mucus Secretion

**Figure 10:**
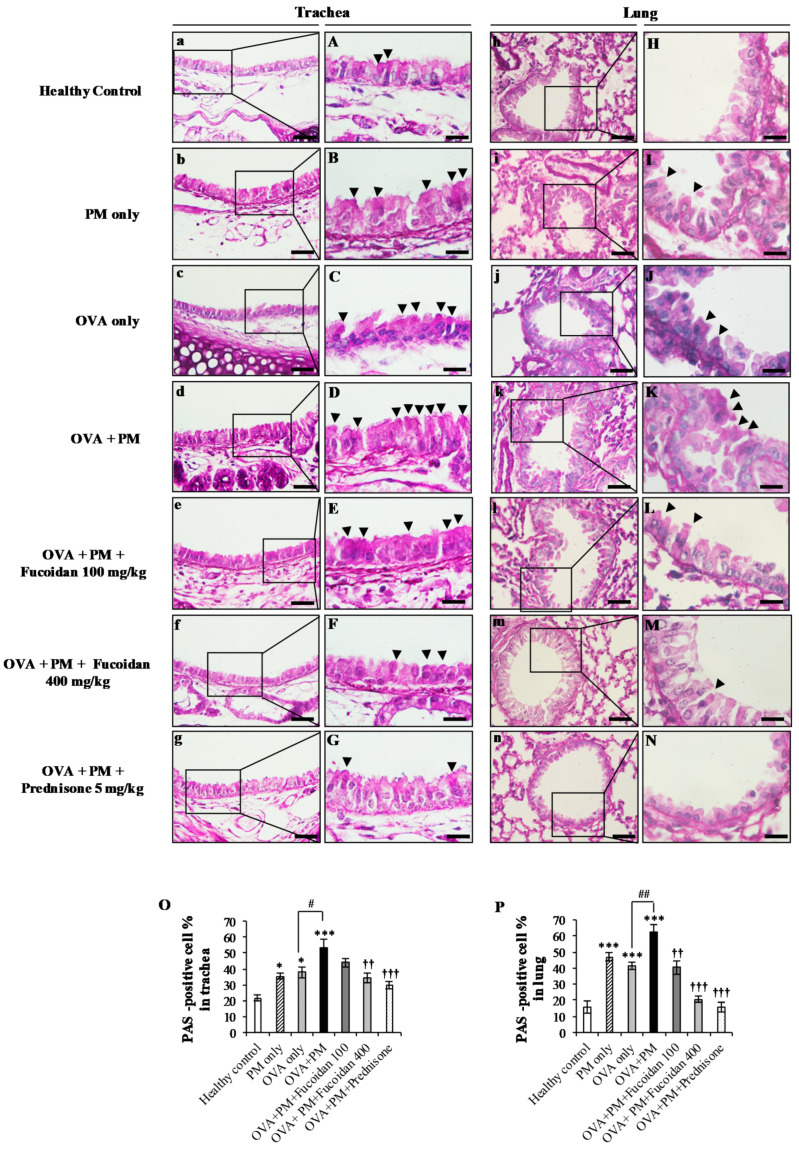
The effect of fucoidans on PAS-positive goblet cells in (**a**–**g**,**A**–**G**) the trachea and lungs (**h**–**n**,**H**–**N**) of PM-exposed mice. PAS-positive cell percentage in (**O**) the trachea and (**P**) the lungs are shown. Arrowheads indicate the mucus secreting goblet cells. Values are expressed as means ± SEM (*n* = 3). * (*p* < 0.05), *** (*p* < 0.0005) represent significant increases compared with the healthy control, and †† (*p* < 0.005), ††† (*p* < 0.0005) represent significant decreases compared with the OVA + PM group. # (*p* < 0.05), ## (*p* < 0.005) represent significant increases compared with the OVA only group. The scale bar of (**a**–**g**) and (**h**–**n**) is 25 µm. The scale bars of (**A**–**G**) is 25 µm, and (**H**–**N**) were captured by an oil immersion lens (magnification ×1000).

One hallmark of asthma is the hyperproduction of mucus in response to inflammatory provocation, and we performed PAS staining to examine goblet cell hyperplasia and mucus secretion (Figure 10). PAS staining of the trachea showed significant increases (by 287.5%, *p* < 0.0005) in goblet cell hyperplasia and mucus secretion in the OVA + PM group compared with the healthy control, confirming the presence of severe airway inflammation. Although the PM only and OVA only groups displayed elevated PAS positive cells compared with the healthy control, the highest PAS positive cell proportion was observed in the OVA + PM group. However, fucoidan treatments attenuated the PAS positive cell proportion in the trachea compared with the OVA + PM group (by 9.1% with a low dose of fucoidan and by 36.7% with a high dose of fucoidan, *p* < 0.005).

In the meantime, PM-challenged and OVA-sensitized mice (OVA + PM group) significantly increased (by 378.0%, *p* < 0.0005) the secretion of mucus and goblet cell hyperplasia in the lungs compared with the healthy control. PM clearly induced PAS positive cells in the lungs (by 49.0%, *p* < 0.005) compared with OVA alone (Figure 10J,P). However, interestingly, fucoidan treatments reduced the number of PAS positive cells compared with the OVA + PM group (by 35.2% (*p* < 0.005) at a low dose and by 66.8% (*p* < 0.0005) at a high dose, respectively) in the lungs. The roles of fucoidans in attenuating goblet cell hyperplasia and mucus secretion validate its therapeutic potential in alleviating PM-exacerbated allergic airway inflammation.

**Figure 11:**
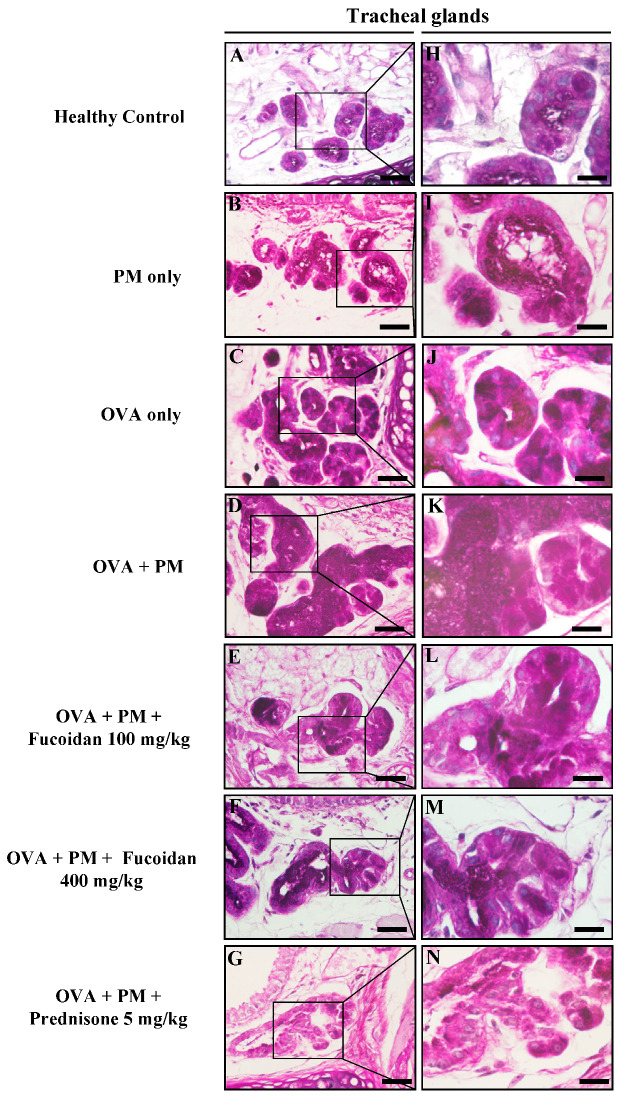
The effect of fucoidans on (**A**–**N**) mucus secretion in submucosal glands in the trachea of PM-exposed mice. Representative figures of (**A**,**H**) the healthy control, (**B**,**I**) PM only, (**C**,**J**) OVA only, (**D**,**K**) OVA + PM, (**E**,**L**) OVA + PM + Fucoidans (100 mg/Kg), (**F**,**M**) OVA + PM + Fucoidans (400 mg/Kg), (**G**,**N**) OVA + PM + Prednisone (5 mg/Kg). The scale bar of (**A**–**G**) is 25 µm, and (**H**–**N**) were captured by an oil immersion lens (magnification ×1000).

Furthermore, PM exposure enhanced mucus secretion with enlarged submucosal mucous glands in OVA-sensitized mice (Figure 11). However, treatments with fucoidans dose dependently reversed the mucus secretion and submucosal gland enlargement (Figure 11E,L for low dose and Figure 11F,M for high dose, respectively).

### 2.11. Fucoidans Attenuated the Levels of the Th2-Derived Cytokine IL-4 and the Epithelial-Cell-Derived Cytokine IL-33

**Figure 12:**
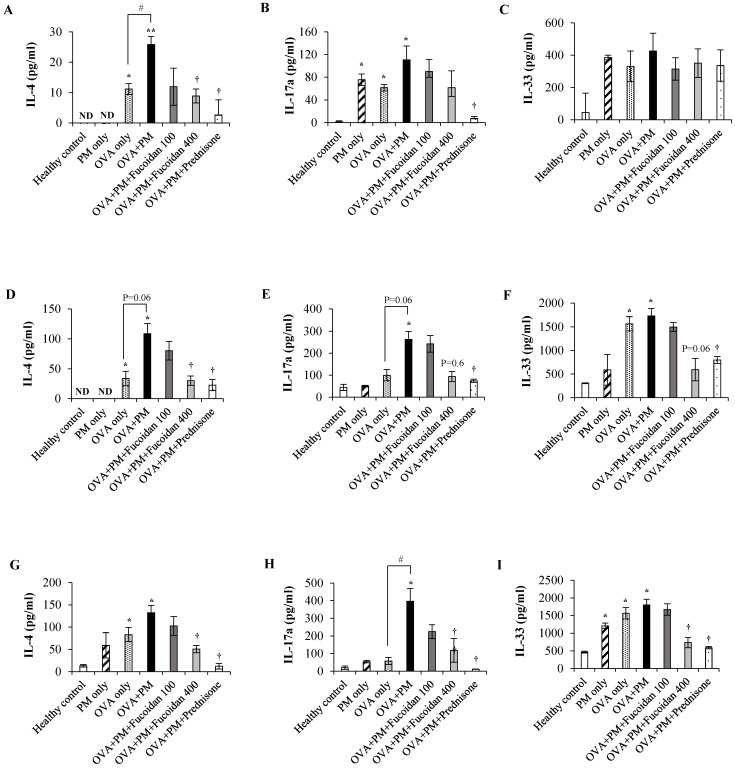
Protein levels in the serum of (**A**) IL-4, (**B**) IL-17a, (**C**) IL-33, BALF: (**D**) IL-4, (**E**) IL-17a, and (**F**) IL-33 and, lung: (**G**) IL-4, (**H**) IL-17a, (**I**) IL-33 were measured using ELISA. Values are expressed as means ±SEM (*n* = 3). * (*p* < 0.05), ** (*p* < 0.005) represent significant increases compared with the untreated control, and † (*p* < 0.05) represents a significant decrease compared with the OVA + PM group. # (*p* < 0.05) represents a significant increase compared with the OVA only group. ND; not detected.

Finally, we evaluated the Th2-derived cytokine IL-4, the Th17-derived cytokine IL-17a, and the epithelial-cell-derived cytokine IL-33 in the serum, Bronchoalveolar Lavage Fluid (BALF), and lungs. The IL-4 level was significantly increased in OVA + PM compared with the healthy control in the serum (Figure 12A, *p* < 0.005), BALF (Figure 12D, *p* < 0.05), and lungs (Figure 12G, *p* < 0.05). PM exacerbated the IL-4 secretion compared with OVA alone, and treatment with high-dose fucoidans attenuated the IL-4 level compared with OVA + PM in the serum, BALF, and lungs (all *p* < 0.05). Interestingly, a high dose of fucoidan was as effective as prednisone, a drug that positively attenuated the PM-exacerbated IL-4 level. The level of IL-17a was also appreciably increased in the serum (Figure 12B), BALF (Figure 12E), and lungs (Figure 12H) of the OVA + PM group compared with the healthy control. Although not significant, however, high-dose fucoidan treatment attenuated the level of IL-17a. Moreover, fucoidans did not attenuate secretion of the epithelial-cell-derived cytokine IL-33 in the serum (Figure 10C). However, we observed a significant increase in the IL-33 level in BALF (Figure 12F) and in the lungs (Figure 12I) of the OVA + PM group compared with the healthy control. Treatment with a high dose of fucoidan attenuated the IL-33 level in BALF and the lungs compared with the OVA + PM group. These results suggest that fucoidans might exert their therapeutic efficacy against PM-exacerbated allergic airway inflammation via attenuating the Th2 immune response.

## 3. Discussion

Epidemiological evidence reveals a clear positive correlation between PM exposure and the pathogenesis of pulmonary diseases such as asthma and chronic obstructive pulmonary disease (COPD). Many studies have identified that exposure to environmental factors such as PM alters the epigenetic landscape in terms of DNA methylation and/or histone modification [19,20]. PM-induced epigenetic modifications can further lead to the development of asthma in infants [19]. Moreover, components in PM, such as heavy metals and polycyclic aromatic hydrocarbons, are known to induce the generation of ROS, the core mediators of inflammation [3]. Since prolonged inflammation is a vital mechanism in the development of pulmonary diseases, PM inhalation and subsequent ROS generation pose a serious risk at all stages of airway injury.

We hypothesized that the use of agents that attenuate PM-exacerbated inflammatory responses might be effective in patients with pulmonary diseases, and we examined the effect of treatment with fucoidans isolated from *U. pinnatifida* sporophylls against PM-exacerbated lung alterations in a mouse model of allergic airway inflammation. Fucoidans isolated from *U. pinnatifida* sporophylls were selected since they have previously been shown to possess anti-inflammatory and anti-allergic effects in several in vitro studies. We wanted to investigate whether the efficacy of fucoidans is still valid in in vivo studies. Prednisone, which is approved for clinical use in patients with allergic asthma, was used as a control anti-inflammatory drug.

Asthma is an inflammatory disease characterized by initial pathological changes such as airway inflammation and mucus hypersecretion. OVA immunization of Balb/c mice is a classic model of allergic airway inflammation [21]. In accordance with previous observations, PM exacerbated OVA-induced airway inflammation and inflammatory cell infiltration, as evidenced by increased primary innate immune cells such as macrophages, eosinophils, neutrophils, and T lymphocytes in the lung tissue [22,23].

CD4^+^ T cells are at the forefront in the pathogenesis of allergic airway inflammation as they specifically identify allergens and initiate an inflammatory response in the respiratory system [24]. Depletion of CD4^+^ T cells attenuated airway hyperresponsiveness and lung eosinophil count in a CD4 antigen induced mouse model [25]. While PM exacerbated the CD4^+^ T cell response in the airways of OVA-sensitized mice, high-dose (400 mg/kg) fucoidan treatment suppressed CD4^+^ T cells similarly to prednisone in this study. Tian et al. also showed that fucoidans attenuate the infiltration of CD4^+^ T cells to skin lesions of 2,4-dinitrochlorobenzene-induced atopic dermatitis mice [26]. It has been reported that sulphated polysaccharides bind with selectins in CD4^+^ T cells and block CD4^+^ T cell function [27]. Indeed, the potential of fucoidans to attenuate CD4+ T cells is probably due to the direct influence of fucoidans on L-selectin expressed on T cells [28]. CD4^+^ T cells exhibit heterogeneous memory responses which can mediate diverse phenotypes like Th2-mediated eosinophilic asthma and Th17-mediated neutrophilic asthma [24]. For instance, Th2 cells secrete cytokines such as IL-4, IL-5, and IL-13 and play important downstream roles in the pathogenesis of asthma [22].

We observed that PM aggravated the IL-4 level in OVA-sensitized mice and, interestingly, fucoidans suppressed the PM-aggravated serum IL-4 level in mice. An earlier study also reported that fucoidans isolated from *U. pinnatifida* sporophylls attenuated the Th2 response via suppressing Th2 cytokines (IL-4, IL-5, IL-13) in an OVA-induced mouse model of asthma [17]. Therefore, we assume that the anti-inflammatory potential of fucoidans exerted previously [29] by increased production of IL-10 mediated the suppression of the Th2 pro-inflammatory cytokine (IL-4). IL-4 is a crucial mediator that induces B cells to switch the allergen specific IgE antibody and carry out eosinophil transmigration and mucus secretion [3]. Consistently, high-dose fucoidan (400 mg/kg) treatment was as effective as prednisone for reducing the serum IgE level, a hallmark of the pathogenesis of Th2-mediated allergic airway inflammation. Moreover, Oomizu et al. revealed a direct influence of fucoidans on suppressing IgE production by blocking Cε germline transcription and nuclear factor κB p52 translocation in B cells [30]. These observations further suggest that fucoidan abrogates PM-exacerbated allergic inflammatory responses by curbing Th2 responses.

IgE, as well as the Th2 response, further activates mast cells. Moreover, PM is known to aggravate mast cell activation and degranulation via enhancing FcεRI-mediated signaling in bone-marrow-derived mast cells [31]. Degranulated mast cells release inflammatory mediators such as histamine, leukotriene, and prostaglandins [31]. These mediators further induce bronchoconstriction and mucus hypersecretion [30]. In our study, fucoidans markedly attenuated mast cell activation as well as mast cell degranulation. A lectin, galectin-9, in fucoidans has been identified to prevent IgE binding to mast cells and mast cell activation [10].

PM-exacerbated IgE secretion and Th2 activation are accompanied by the activation of eosinophils, and the recruitment of eosinophils in the airway further release eosinophil cationic proteins and eosinophil peroxidases, as well as opening influx channels for cytotoxic molecules [32]. Therefore, the accumulation of eosinophils might lead to further development of a severe allergic inflammatory response and allergic asthma. Our results demonstrate that fucoidans attenuate PM-exacerbated eosinophil accumulation in trachea and lung tissues. High-dose fucoidan (400 mg/kg) treatment was as effective as prednisone for suppressing PM-exacerbated eosinophil infiltration in the airways. It is reported that the polysaccharide fucoidan binds to L-selectin on eosinophils and adversely affects the eosinophils rolling to the sites of inflammation in guinea-pig skin [33].

On the contrary, we reported that PM exacerbates the Th17 immune response as well as Th2 response [22]. Fucoidan treatment attenuated PM-exacerbated Th17 cytokine IL-17a secretion, and fucose in fucoidans might be responsible for attenuating their secretion [34]. It is important to note that our fucoidans (isolated from sporophylls of *U. pinnatifida*) contain a high fucose content compared with other fucoidans. Activated IL-17a can further infiltrate neutrophils to the site of inflammation and accordingly, a high dose of fucoidan markedly attenuated the neutrophil influx in the airways. Therefore, it is speculated that high-fucose-containing fucoidan attenuates the PM-exacerbated Th17 response in the face of attenuated IL-17a and neutrophil influx, thereby acting as a protective agent against allergic airway inflammation.

Consistent with previous reports [35,36], we observed that our OVA model developed a Th2-predominant (increased IL-4 secretion) as well as a Th17-predominant (increased IL-17a secretion) immune response with significantly enhanced pulmonary eosinophil and neutrophil influx. These results verify that we successfully established a mixed eosinophilic and neutrophilic phenotype in the Th2/Th17-predominant allergic airway inflammation model. Moreover, PM exacerbated the OVA-induced Th2/Th17-predominant allergic airway inflammation.

It was recently discovered that epithelial cells also play a fundamental role in initiating different forms of asthma (Th2/Th17/mixed) with altered functional barrier properties [37]. Different epithelial cell types in the airways are involved in the initiation and exaggeration of the Th2 immune response mainly via epithelial derived alarmins such as IL-33 and thymic stromal lymphopoietin (TSLP) [37,38]. Th2-mediated asthma mechanisms (Th2 inflammatory responses and epithelial barrier alterations) have been reported to be initiated in parallel but independently. In our results, we demonstrated that a high dose of fucoidan attenuates the IL-33 level in BALF and the lungs as effectively as prednisone. The potential of fucoidans to attenuate IL-33 might also be responsible for the attenuated Th2 response in asthmatic mice. Moreover, the chemoattractant secreted by activated NF-ҡB signaling in epithelial cells recruits neutrophils to the lungs, mediating Th17-associated neutrophilic asthma [39]. The altered phenotype of epithelial cells further promotes degradation of tight junctions and barrier permeability, sustaining the penetration of exogenous materials [37].

Exogenous materials such as PM can increase the number of host defense phagocytic cells and macrophages in the host. Recently, fine particles were observed as round dark granules in the macrophages of guinea pigs that inhaled PM [40]. Macrophages are also induced by Th2-activated IL-4, and induced macrophages, in turn, produce nitric oxides and inflammatory mediators such as cycloxygenase-2, induce mucus secretion, and worsen the symptoms of asthma [41]. In our study, fucoidans subdued PM-exacerbated macrophages in the trachea and lungs. In support of our findings, Kim et al. (2010) discovered that fucoidans from *U. pinnatifida* sporophylls suppressed the secretion of inflammatory cytokines IL-1β and TNF-α in LPS-induced RAW264.7 cells [16]. In addition, Keiko et al. reveled that l-fucose blocks macrophage activation under oxidative stress conditions [42]. Our fucoidans, which are rich in l-fucose, were further shown to be effective against allergic airway inflammation conditions.

Despite having significant pulmonary allergic inflammation, we observed goblet cell hyperplasia and increased mucus secretion in PM-exposed mice, and we believe this is a consequence of the PM-exacerbated activation of the Th2/Th17 response and macrophage infiltration [41]. Accumulation of a mucus plug in the airways is observed in patients with lung inflammatory diseases, and this leads to obstruction and failure of respiration. Effective treatment for mucus hypersecretion is an unmet medical demand in curtailing asthma discomfort [43]. Interestingly, fucoidans were found to attenuate goblet cell hyperplasia, enlargement of submucosal glands, and mucus hypersecretion. It is conceivable that fucoidans effectively repress PM-aggravated mucus hypersecretion. These observations suggest that fucoidans isolated from *U. pinnatifida* can be effective natural candidates to act against PM-exacerbated airway allergic inflammatory responses.

## 4. Materials and Methods

### 4.1. Reagents and Kits

OVA, prednisone, acetyl-β-methylcholine, Congo red, eosin, toluidine blue, periodic acid of Schiff, leupeptin, phenylmethylsulfonyl fluroride (PMSF), sodium ortho-vanadate (NOV), aprotinin, and malondialdehyde (MDA) were purchased from Sigma (St. Louis, MO, USA). Hematoxylene was purchased from Dako (Carpinteria, CA, USA). Methyl green was purchased from Fluka^TM^ (São Paulo, Brazil). Alum adjuvant was purchased from Thermo Fisher Scientific (Rockford, IL, USA). Thiobarbituric acid was purchased from Alfa Aesar (Ward Hill, MA, USA). Trichloroacetic acid was purchased from Merck (Darmstadt, Germany). Bradford reagent was purchased from Bio-Rad (Richmond, CA, USA). The ELISA kit for measuring total IgE was purchased from Bethyl Lab (Montgomery, TX, USA). ELISA kits for measuring OVA-specific IgE, OVA-specific IgG2a, IL-4, IL-6, and IL-17a were purchased from BioLegend (San Diego, CA, USA). ELISA kits for measuring OVA-specific IgG1 was purchased from Cayman chemicals (Ann Arbor, MI, USA). The Mouse IL-33 ELISA kit was purchased from Invitrogen (Thermo Fisher Scientific, Waltham, MA, USA). The DNA/RNA oxidative damage ELISA kit was purchased from Cayman Chemicals (Ann Arbor, MI, USA). The Diff Quick staining kit was purchased from Sysmex Corporation (Kobe, Japan). Xylene and ethanol were purchased from OCI Chemicals (Seoul, S. Korea). The anti-malondialdehyde antibody was purchased from abcam (Cambridge, UK). The mouse Gr-1 antibody was purchased from R&D Systems (Minneapolis, MN, USA). The purified anti-mouse F4/80 antibody was purchased from BioLegend (San Diego, CA, USA). The CD4 antibody was purchased from Novus Biologicals (LLC, USA). The fucoidans were supplied by Haewon Biotech Co., Ltd. (Seoul, Korea).

### 4.2. Particulate Matter

PM from the certified reference material (CRM) No. 28 urban aerosol, developed and certified from National Institute for Environmental Studies (NIES), Japan, was used in this study. The majority of the PM was <2.5 µm in diameter, and a large proportion of the PM sample contained polycyclic aromatic hydrocarbons (PAH) and heavy metals such as Pb and Ba [44].

### 4.3. Animals

Female Balb/c mice (20–25 g, 7–8 weeks old), purchased from OrientBio, Sungnam, Korea, were housed in a conventional animal facility and fed an NIH-07-approved diet along with water ad libitum. Animal experiments were performed in accordance with the Guidelines for the Care and Use of Laboratory Animals of the Institutional Ethical Committee of Jeju National University.

### 4.4. Experimental Design

**Figure 13:**
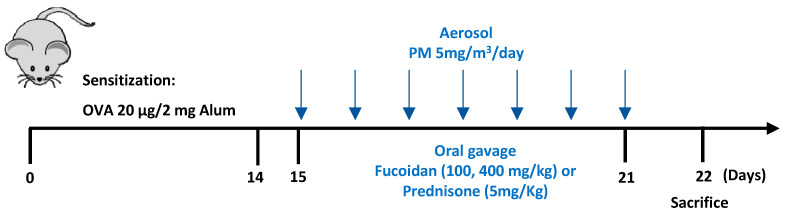
Experimental plan for PM-exacerbated allergic airway inflammation in OVA-sensitized mice.

The PM exacerbated mouse model of allergic airway inflammation was utilized following a protocol described previously [22] with minor modifications. Mice (*n* = 7) were randomly divided into the following groups: healthy control, PM only, OVA only, OVA + PM, OVA + PM + Fucoidans (100 mg/kg), OVA + PM + Fucoidans (400 mg/kg), and OVA + PM + Prednisone (5 mg/kg). Mice in the OVA induction groups (OVA only, OVA + PM, OVA + PM + Fucoidans, OVA + PM + Prednisone) were sensitized with OVA (10 µg) dissolved in 200 µL saline and 2 mg Al(OH)_3_ by intraperitoneal (i.p) injections. Two weeks after the sensitization, mice in groups other than the healthy control and OVA only groups were nebulized with PM (5 mg/m^3^, 30 min/day) daily for 7 consecutive days. Together with PM exposure, the PM exposed mice were orally gavaged with either a low dose (100 mg/kg) or high dose (400 mg/kg) of fucoidans or prednisone (5 mg/kg) suspended in saline. Other groups were orally administered with saline (Figure 13).

### 4.5. Measurement of Malondialdehyde (MDA) Level in Serum

The degradation of lipids is a useful marker for oxidative stress, which further increases the production of free radicals. MDA is a critical end product of polyunsaturated lipids that have undergone oxidative attack. We measured the MDA level in the serum of mice using a lipid peroxidation assay kit following the manufacturer’s instructions. MDA activity was measured at the micro molar level based on the standard curve using a colorimetric assay.

### 4.6. Thiobarbituric Acid Reactive Substance (TBARS) Assay

We measured the lipid peroxidation of the lungs as previously described [45]. In brief, we homogenized the lung tissues using taco™Prep bead beater (GeneReach Biotechnology Co., Taichung City, Taiwan) using RIPA buffer containing protease inhibitors (2 mM Na3VO4, 1 mM PMSF, 10 μg/mL aprotinin, 10 μg/mL leupeptin). Obtained homogenate was centrifuged (at 3000× *g* for 10 min at 4 °C), and the protein concentration in the supernatant was measured by Bradford protein assay method. An equal amount of protein was mixed with 10% (*w*/*v*) trichloroacetic acid and allowed to incubate for 10 min (at 4 °C). After centrifuging at 2000× *g* (15 min, 4 °C), the resulting supernatant was reacted with equal volume of 0.67% (*w*/*v*) thiobarbituric acid in a heating block for 10 min. The supernatant was separated by centrifuging at 2000× *g* (15 min at 4 °C), and the absorbance of the supernatant was measured at 532 nm using a microplate reader. The malondialdehyde (MDA) content in the samples was measured with the predetermined MDA standard curve.

### 4.7. Methacholine Test for Airway Hyper-Responsiveness

After 24 h of the final challenge with PM the mice were nebulized with different concentrations of methacholine (0, 12.5, 25, 50 mg/mL) for 10 min in a whole body chamber. Immediately after each nebulization, the enhanced pause (Penh) was measured in a plethysmography chamber (Allmedicus, Anyang, Korea). The readings were averaged for a 3 min period.

### 4.8. Bronchoalveolar Lavage Fluid (BALF) Collection and Differential Counting

BALF was collected from euthanized mice as previously described [22]. In brief, we instilled 1ml of DPBS into the lungs (intra-tracheal) using a cannula and gently collected BALF. The collected BALF was centrifuged (300× *g*, 5 min, at 4 °C), and the supernatant was separated for cytokine analysis. The cells were fixed in methanol and the smears were prepared on glass slides. The smears were stained with a Diff Quick staining kit and the cell compositions of macrophages, eosinophils, neutrophils, lymphocytes, and basophils were determined using a microscope with a Olympus DP-72 microscope camera (Olympus, Tokyo, Japan).

### 4.9. Histological Examination of Trachea and Lung

Mice were sacrificed one day after final exposure to PM (day 22), and lung and trachea sections were fixed in 10% formalin. After fixation, samples were embedded in paraffin and sectioned (3 µm) onto coated glasses.

Tissue sections were stained with hematoxylene and eosin (H&E) and observed under a light microscope (Olympus, Tokyo, Japan) for histopathological examinations. Inflammation in trachea and lung sections were scored as follows [46]: 0, normal; 1, few inflammatory cells infiltrated; 2, observed inflammatory cells one cell layer deep; 3, observed inflammatory cells 2–4 cell layers deep; and 4, infiltrated inflammatory cells of more than four cell layers deep.

Infiltration of eosinophils in the lung and trachea tissue sections was confirmed by using Congo red staining. Deparaffinized tissues were immersed in Congo red working solution for 30 min. Sections were then washed in running tap water and counterstained with 0.5% methyl green. Stained sections were observed under a microscope, and representative areas were captured using an Olympus DP-72 microscope camera (Olympus, Tokyo, Japan). The number of Congo red stained eosinophils per site from at least three sites of each section (3 sections were taken from each mouse) was quantified using ImageJ (v1.46) software.

Mast cell infiltration in the trachea was evaluated using toluidine blue staining. Deparaffinized trachea sections were immersed in 0.5% toluidine blue solution and counterstained with 0.5% methyl green. Stained sections were observed under a microscope, and representative areas were captured using an Olympus DP-72 microscope camera (Olympus, Tokyo, Japan). The number of mast cells per site and the number of degranulated mast cells per site from at least three sites of each section (3 sections were taken from each mouse) were quantified using ImageJ (v1.46) software.

Trachea and lung sections were stained with periodic acid-Schiff (PAS) and counterstained with 0.5% methyl green to evaluate the goblet cell metaplasia and mucus secretion. The PAS-positive cell percentage from at least three sites of each section (3 sections were taken from each mouse) was quantified using ImageJ (v1.46) software.

### 4.10. Immunohistochemical Analysis of Trachea and Lung

Trachea and lung sections on glass slides were de-paraffinized in xylene series and rehydrated in ethanol series. Nonspecific binding was blocked by immersing tissues in 0.3% hydrogen peroxide with rabbit or horse serum for 30 min. Following the blocking, tissues were incubated with either mouse Gr-1 antibody (1:200) or anti-mouse F4/80 antibody (1:200), anti CD4 antibody (1:400) or MDA antibody (1:400) overnight at 4 °C. After washing, tissues were incubated with biotinylated anti-rat IgG or anti-rabbit IgG for 45 min followed by treatment with an avidin-biotin peroxidase complex using the Vectastain Elite ABC kit (Vector Inc., Burlingame, CA, USA). HRP binding sites were detected with 3, 3′-diaminbenzidine (DAB, Vector Inc.). Images were captured using an Olympus DP-72 microscope camera (Olympus, Tokyo, Japan). The number of positive cells per site from at least three sites of each section (3 sections were taken from each mouse) was acquired with ImageJ (v1.46) software.

### 4.11. Enzyme-Linked Immunosorbent Assay (ELISA) Analysis

Blood was collected from anaesthetized mice on day 22 via cardiac puncture using a heparinized syringe. Serum was separated from blood cells by centrifuging collected blood at 12,000 rpm for 10 min at 4 °C. The serum levels of IL-4, IL-17a, IL-33, 8-OHdG-acethycholinesterase conjugate (DNA/RNA oxidative damage tracer), IgE, OVA-specific IgE, OVA-specific IgG1 (1:2000 diluted serum), and OVA-specific IgG2a were analyzed using the respective ELISA kits following the manufacturers’ recommendations.

After blood collection, mice were sacrificed and 1 mL of DPBS was instilled gently into the lungs of mice using a canula, and BALF was collected as previously mentioned [22]. In brief, we centrifuged the collected BALF (12,000 rpm, at 4 °C) for 10 min and the supernatant was analyzed for the levels of IL-4, IL-17a, and IL-33 using the respective ELISA kits.

We homogenized the lung tissues using the taco™Prep bead beater (GeneReach Biotechnology Co.) using RIPA buffer containing protease inhibitors (2 mM Na3VO4, 1 mM PMSF, 10 μg/mL aprotinin, 10 μg/mL leupeptin). The obtained homogenate was centrifuged (at 3000× *g* for 10 min at 4 °C), and the levels of IL-4, IL-17a, and IL-33 in the supernatant of the lung homogenate were analyzed using the respective ELISA kits.

### 4.12. Statistical Analysis

One-way analysis of variance (ANOVA) followed by Tukey’s multiple comparison was performed to compare the mean values among groups. A *p* value <0.05 was considered statistically significant.

## 5. Conclusions

Taken together, our results suggest that a high dose (400 mg/Kg) of fucoidans from *U. pinnatifida* sporophylls notably blocks ambient PM (<2.5 µm) exacerbated airway inflammation and mucus hypersecretion in a PM-induced mouse model of allergic airway inflammation. A high dose (400 mg/Kg) of fucoidans was found to be similarly effective to prednisone, a widely used immunosuppressive corticosteroid for attenuating the PM-exacerbated MDA level, inflammatory cell activation (macrophages, eosinophils, granulocytes, CD4 T cells, mast cells), and mucus hypersecretion in this study. Therefore, we conclude that fucoidans can be effective natural candidates for use against fine PM (<2.5 µm) exacerbated airway allergic inflammatory responses.
